# Editorial: Emerging zoonotic diseases: understanding and mitigating risks at animal-human interfaces

**DOI:** 10.3389/fvets.2026.1799435

**Published:** 2026-04-02

**Authors:** Christopher Hamilton-West, Francisca Di Pillo, Klaas Dietze, Jose L. Gonzalez

**Affiliations:** 1 Departamento de Medicina Preventiva Animal, Facultad de Ciencias Veterinarias y Pecuarias, Universidad de Chile, Santiago, Chile; 2 Núcleo de Investigación en One Health, Facultad de Medicina Veterinaria y Agronomía, Universidad de Las Américas, Campus Providencia, Santiago, Chile; 3 Institute of International Animal Health/One Health, Friedrich-Loeffler-Institut, Federal Research Institute for Animal Health, Greifswald-Insel Riems, Germany; 4 Epidemiology Group at Wageningen Bioveterinary Research, Wageningen, Netherlands

**Keywords:** disease prevention, infectious diseases, interfaces, One Health, zoonoses

Zoonotic diseases remain a persistent and evolving threat to human health, animal health and welfare, food safety, and socioeconomic stability. Interactions among wildlife, domestic animals, and people are being reshaped by land-use change and habitat fragmentation, urban expansion, transitions in animal production systems, wildlife trade, climate variability, and the globalization of travel and commerce. Together, these drivers alter host density, species mixing, and environmental conditions, thereby increasing spillover opportunities and complicating prevention and control.

Mitigating these risks requires integrative approaches that recognize the interdependence of human, animal, and environmental health. The One Health framework provides a practical foundation for this integration by connecting veterinary science, public health, microbiology, ecology, and the social sciences, and by encouraging the co-design of surveillance and interventions with communities and stakeholders.

This Research Topic, “*Emerging Zoonotic Diseases: Understanding and Mitigating Risks at Animal–Human Interfaces*,” brought together a substantial set of original research articles, reviews, and case reports. A defining feature of the collection is its broad geographic reach (Figure 1), spanning diverse socio-ecological contexts and production systems across Africa, Asia, the Middle East, and the Americas. This breadth highlights both shared drivers of zoonotic risk and the importance of locally grounded evidence for prevention and control.

**Figure 1:**
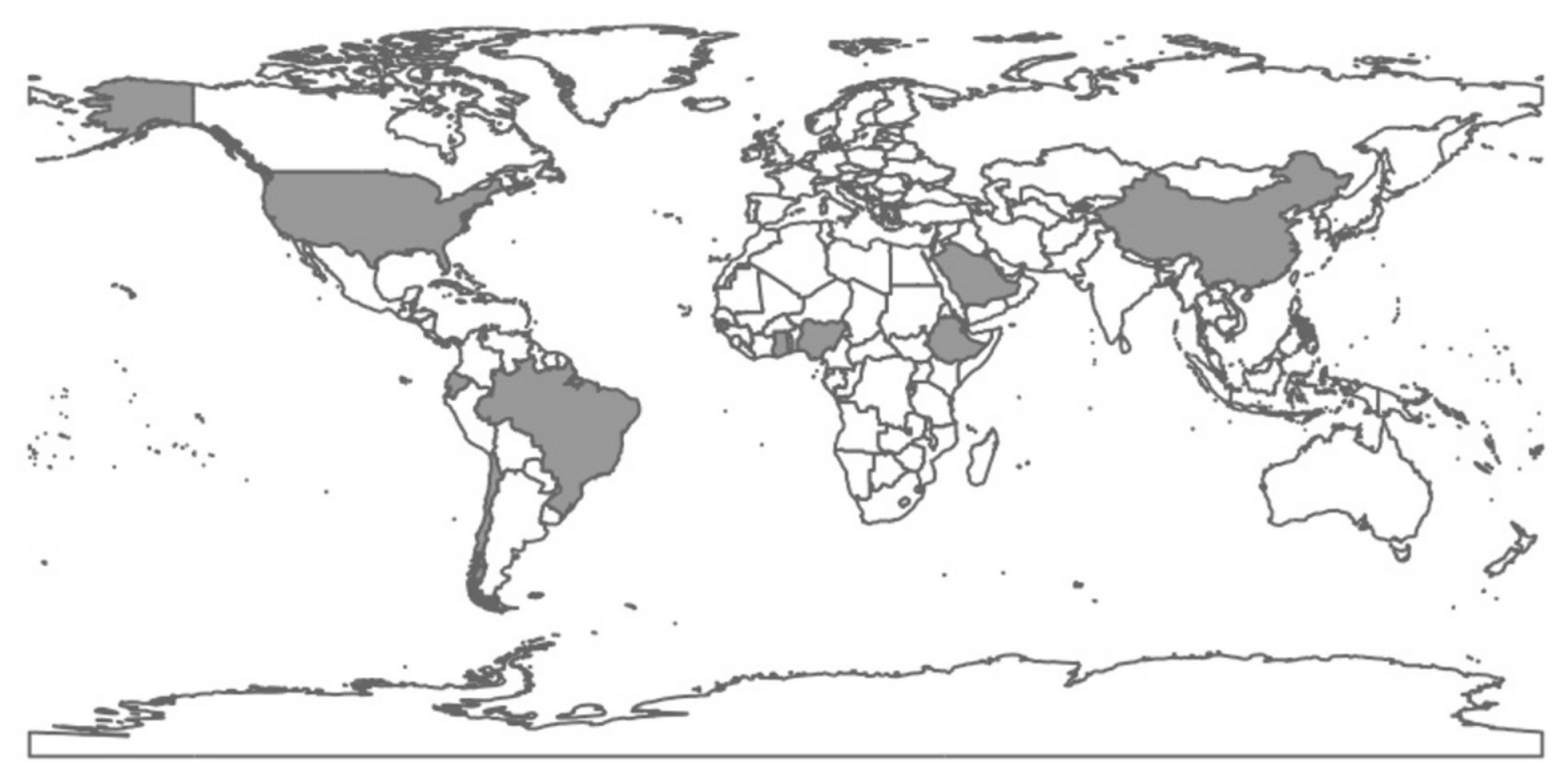
Geographic distribution of manuscripts received for the Research Topic “Emerging zoonotic diseases: understanding and mitigating risks at animal–human interfaces.”

## Thematic synthesis of contributions

### Bacterial zoonoses and antimicrobial resistance

Several studies examine bacterial pathogens and antimicrobial resistance (AMR) at the animal–human interface. These include epidemiological research on psittacosis in China (Wen et al.); investigations in Saudi Arabia describing *Hyalomma dromedarii* ticks infesting camels and carrying antimicrobial-resistant bacteria (Aljasham et al.); and studies in Brazil documenting *Staphylococcus pseudintermedius* in dogs and their owners (Martins et al.), pointing to potential household- and clinic-level transmission pathways. Complementing these, comparative genomic analyses of Shiga toxin-producing *Escherichia coli* in humans and cattle in Chile (Martínez et al.) provide insights into host-associated adaptations relevant to understanding the potential for cross-species transmission and informing control measures.

### Viral zoonoses and spillover risk

Viral pathogens are prominently featured. Case-based and field investigations document rabies virus transmission across domestic animal species (Odoom et al.), including a reported dog-to-bovine event in Ghana. Other studies examine exposure routes at close contact interfaces, such as canine exposure to human papillomavirus in China (Liu et al.). Meanwhile, modeling research from the United States evaluates the zoonotic transmission risk posed by swine influenza A variants (Pittman Ratterree et al.), demonstrating how quantitative approaches can aid preparedness by assessing potential exposure and transmission scenarios.

### Often overlooked fungal and parasitic zoonoses

The collection expands the focus beyond bacteria and viruses by including fungal and parasitic pathogens with clear One Health relevance. Studies from Ecuador report a high prevalence of *Histoplasma capsulatum* in bats and pigeons (Mora-Jaramillo et al.) in environments associated with human histoplasmosis, emphasizing the need to consider peri-domestic wildlife and synanthropic species as exposure routes. Additional research examines guinea pigs raised as livestock as incidental hosts for *Toxoplasma gondii* and influenza A viruses (Salas-Rueda et al.), highlighting interfaces that may be missed in traditional surveillance methods. A detailed case report from Chile describing feline sporotrichosis progressing to sepsis (Cartes et al.) underscores the clinical severity of emerging fungal infections and underscores the importance of prompt recognition and safe handling to prevent zoonotic exposure.

### Endemic zoonoses, livelihoods, and context-specific risks

Several studies focus on endemic zoonoses in resource-limited, pastoral, or high-contact environments, where zoonotic risk intersects with livelihoods and health system limitations. Epidemiological analyses from southern Ethiopia examine *Mycobacterium tuberculosis* complex infections in cattle and humans (Mohammed et al.), illustrating how shared environments and occupational exposure influence transmission patterns. Social and behavioral research from Nigeria (Muhinda et al.) assesses knowledge, attitudes, and practices related to mpox and other zoonoses among actors in bushmeat value chains, emphasizing that risk reduction strategies must be practical within local economic contexts and aligned with trusted communication channels.

### Methods, evidence synthesis, and translation into practice

Methodological and synthesis-focused contributions enhance the translational value of the Research Topic. A global field guide for bat sampling (Islam et al.) offers standardized methods for eco-epidemiological investigations, making results more comparable across studies and supporting safer, more consistent field practices. Additionally, a systematic review and meta-analysis (Huber et al.) evaluating biosecurity measures to reduce *Salmonella* spp. and hepatitis E virus on pig farms provides evidence-based guidance for practical interventions. Together, these contributions help bridge the gap between knowledge generation and implementation of measures to reduce risk in real-world settings, while also highlighting where evidence is limited and where future research should focus.

## Geographic breadth and One Health implications

Across these themes, the collection demonstrates that “the interface” is not a single setting but a range of systems, ranging from households and veterinary clinics to intensive farms, wildlife peri-domestic ecotones, and informal value chains. Despite variations in local drivers and constraints, several common messages emerge. First, zoonotic risk often concentrates where interfaces become denser and more mixed, environmental disturbance increases, or supply chain connectivity is higher. Second, surveillance approaches are more comprehensive and likely to be more sensitive when designed multisectorally, linking animal and human health data, incorporating ecological observations, and combining laboratory diagnostics with epidemiological analysis. Third, successful prevention and control efforts depend on practicality: interventions must align with the social, economic, and governance realities of the systems in which they are applied, and be supported by community engagement and credible, culturally suitable risk communication.

Taken together, the contributions in this Research Topic show how One Health research can shift from merely describing interfaces to identifying practical leverage points, enhancing detection of zoonotic threats, clarifying exposure pathways, and guiding interventions that lower risk while accounting for social and economic constraints.

## Looking forward

The strong global engagement and the diversity of themes represented in this Research Topic highlight the continued importance of interdisciplinary investigations that address zoonotic risks at animal–human interfaces. Ongoing progress will rely on continued investment in One Health surveillance platforms, laboratory and analytical skills, and policy implementation that adapts to local contexts. We hope this collection will be a useful resource for researchers, practitioners, and decision-makers, and will encourage further interdisciplinary efforts to better understand and more effectively reduce zoonotic risks at animal–human interfaces.

